# Delirium screening tools in the emergency department: A protocol for systematic review and meta-analysis

**DOI:** 10.1097/MD.0000000000024779

**Published:** 2021-02-26

**Authors:** Qian Zhang, Sheng Li, Meixi Chen, Qiuyu Yang, Xiao Cao, Long Ge, Baoshan Di

**Affiliations:** a School of Nursing, Lanzhou University; b The First People's Hospital of Lanzhou City; c The First Clinical Medical College, Lanzhou University; d Department of Social Medicine and Health Management, School of Public Health, Lanzhou University; e The First Hospital, Lanzhou University, Lanzhou, China.

**Keywords:** delirium, meta-analysis, screening, systematic review

## Abstract

**Background:**

Delirium is a common type of acute brain dysfunction among emergency department (ED) patients. The prevalence of delirium in the ED is up to 40%. Although screening instruments used to identify delirium have been developed, it is unclear which tool is the most accurate in the ED. To address this challenging, we systematically examine the accuracy of delirium screening tools used to assess the ED patients.

**Methods:**

This study has been registered at the International Platform of Registered Systematic Review and Meta-Analysis Protocols (INPLASY), and the registration number is INPLASY202110041. We will search the PubMed, EMBASE, PsycINFO, and the Cochrane Library. Studies involving patients which compared diagnostic instruments with the criteria in Diagnostic and Statistical Manual of Mental Disorders (DSM) as a reference standard will be included. We will use STATA 15.1 and MetaDiSC to make careful analysis of the results. The quality of included studies will be assessed using the Quality Assessment of Diagnostic Accuracy Studies (QUADAS)-2 scale.

**Results:**

In this study, the accuracy of different screening methods among ED patients is assessed by a high-quality synthesis. The number of tools available for screening delirium in the ED, the information of studies including the countries, the study design, the sample size and the characteristic of studies, the quality of the studies and the results of meta-analysis. The systematic review and meta-analysis will be published in a peer-reviewed journal.

**Conclusion:**

According to the conclusion of the systematic review, evidence will be provided to judge which screening method is the best for the ED patients. The results will bring better understanding of screening methods in the ED and highlight gaps for future research.

## 1 Introduction

Delirium is a mental fluctuation syndrome with a drastic change in cognition, consciousness level, and fall in attention level.^[1,2]^ In the emergency department (ED), the prevalence reported as high as 40%, and 8% to 25% of old patients in ED present with delirium.^[3,4]^ Patients affected delirium intended to have poor outcomes, including longer hospital stays, a higher rate of hospital-acquired complications, and increased mortality.^[5–7]^ In the United States, 1-year healthcare costs associated with delirium are estimated to be $38 billion.^[8]^ Although delirium is common and associated with serious adverse consequences, there is still 3 out of 4 patients missed delirium detection by clinical healthcare staff.^[9,10]^ Because of the specificity of the ED, screening for delirium is more difficult. The high volume of patients and tense time demands on providers make screening for delirium more difficult in the ED.^[11]^ Studies have shown that screening for delirium in the ED is rare done. Emergency physicians miss about 1.2 million cases of delirium each year in the United States.^[12]^ Although guidelines for the management of delirium have recommended that detection should be performed as early as possible.^[13]^ It was only rarely done because delirium monitoring was often complicated and time-consuming.^[14]^ Delirium screening is still a challenge for the ED staff. As the gateway for a significant proportion of hospital admissions, improving our understanding of delirium in the ED is crucial to improving the quality of care delivered to the patient.^[15]^ Therefore, this indicates the need for screening tools. Clinical practice guidelines recommend that an effective delirium assessment tool is an important part of delirium detection.^[16]^ Accurate screening tools can identify high-risk patients to reduce or prevent the occurrence of delirium and reduce the burden of delirium.^[17]^

Currently, indicators for delirium screening and diagnosis have not been uniformly recognized.^[18]^ Different screening tools have a variety of sensitivities and specificities.^[19]^ The time used to complete the assessments also adds to the complexity of delirium detection.^[20]^ Different guidelines provide different recommendations. According to National Institute for Health and Care Excellence (NICE), the short Confusion Assessment Method (short CAM) should be routinely used in the acute hospital settings to diagnosis delirium.^[21]^ The Scottish Intercollegiate Guidelines Network (SIGN) recommends that the 4AT (Arousal, Attention, Abbreviated Mental Test 4, Acute change) tool shall be used for identifying patients with probable delirium in emergency department and acute hospital settings.^[22]^ Although many assessment tools are already in use, what assessment tools are most effective in ED patients remains unknown.

So far, several systematic reviews have been conducted to determine which is the best for delirium screening in the ED, but they did not provide a pooled analysis of the accuracy of existing assessment tools. Ewan et al summarized the results for delirium assessment and concluded that there is variability in screening methodology, the procedures to obtain consent and the methodological quality.^[23]^ A validated screening method is urgently needed to identify delirium in the early time. Michael et al concluded that there is still a lack of validated delirium screening tools in the ED.^[18]^ José et al conducted a systematic search and found that the Confusion Assessment Method for the Intensive Care Unit (CAM-ICU) is the most widely used instrument, but not the most suitable for the ED.^[24]^ The best tool for delirium screening in the ED remains to be determined

Therefore, this paper was designed to assess the screening accuracy of different assessment tools for ED patients by using a meta-analysis approach, and grade different methods of assessment using the superiority index.

## 2 Methods and analysis

### 2.1 Registration

This study has been registered at the International Platform of Registered Systematic Review and Meta-analysis Protocols (INPLASY.COM), and the registration number is INPLASY202110041. This systematic review scheme shall be subject to Preferred Reporting Items for Systematic review and Meta-Analysis Protocols 2015 statement (PRISMA-P).^[25]^

### 2.2 Eligibility criteria

Studies meeting the following criteria will be included:

1. population limited to ED patients;
2. index tests that included at least one delirium assessment tool for diagnosed patients (e.g., CAM, 4AT) which was compared with the reference standards (Diagnostic and Statistical Manual of Mental Disorders [DSM]).
3. adequate information for the calculation of true positive (TP), false positive (FP), true negative (TN), and false negative (FN) values; and
4. cohort or cross-sectional designs.

We will not limit the language or year of publication. We will exclude editorials, commentaries, as well as pilot, case report, and duplicated studies.

### 2.3 Search methods to identify the researches

#### 2.3.1 Electronic materials

PubMed, PsycINFO, EMBASE, and the Cochrane Library will be used from the beginning of the study to January 2021. Under the guidance by LG, an experienced evidence-based medicine researcher, LG and QZ will develop the search strategies. The search terms were “delirium,” “acute confusion,” “diagnosis,” “sensitivity,” and “specificity.” The details of the PubMed search are provided in Table 1. The duplicates identified after the database search will be removed. Potential researches will be identified by searching the references of related systematic reviews and meta-analysis.

**Table 1:** Search strategy for PubMed.

#	Search Terms
#1	“Delirium”[Mesh]
#2	Search (((((((((((((((((delirium[Title/Abstract]) OR deliri^∗^[Title/Abstract]) OR “acute confusion”[Title/Abstract]) OR “acute organic psychosyndrome”[Title/Abstract]) OR “acute brain syndrome”[Title/Abstract]) OR “acute brain dysfunction”[Title/Abstract]) OR “acute brain failure”[Title/Abstract]) OR “organic psychosyndrome”[Title/Abstract]) OR “metabolic encephalopathy”[Title/Abstract]) OR “psycho-organic syndrome”[Title/Abstract]) OR clouded state^∗^[Title/Abstract]) OR “clouding of consciousness”[Title/Abstract]) OR “exogenous psychosis”[Title/Abstract]) OR “toxic psychosis”[Title/Abstract]) OR “toxic confusion”[Title/Abstract]) OR obnubilat^∗^[Title/Abstract]) OR “mental confusion”[Title/Abstract]) OR “clouding of consciousness”[Title/Abstract]
#3	#1 OR #2
#4	“Sensitivity and Specificity”[Mesh]
#5	((Sensitivity[Title/Abstract]) OR Specificity[Title/Abstract]) OR (Sensitivity[Title/Abstract] AND Specificity[Title/Abstract])
#6	#4 OR #5
#7	(((accuracy[Title/Abstract]) OR diagnos^∗^[Title]) OR screen^∗^[Title]) OR detect^∗^[Title]
#8	#6 OR #7
#9	#3 AND #8

#### 2.3.2 Study records

**Figure 1:**
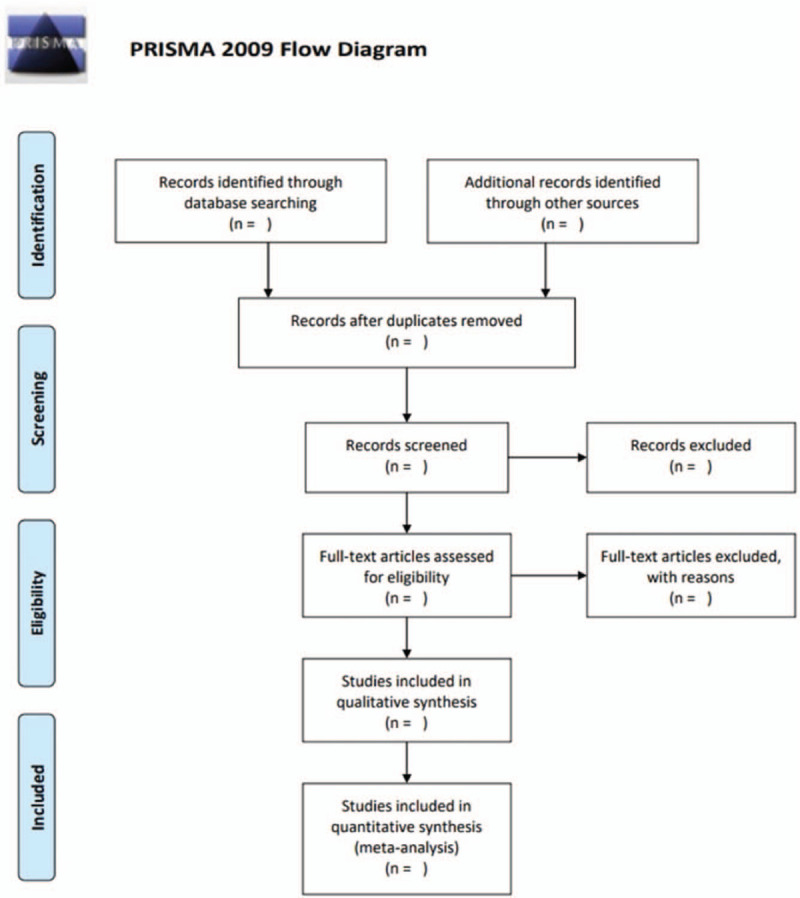
Flow chart of the study selection procedure. Figure 1 shows the flow chart of the study selection procedure according to the Preferred Reporting Items for Systematic Reviews and Meta-Analyses (PRISMA) Statement.

EndNote X9 will be used to manage the initial search records. After removing duplicate records, the remaining records will be imported to Rayyan, a free mobile app and web for systematic reviews.^[26]^ The titles and abstracts of all records identified will be screened independently by teams of 2 reviewers (MXC, SL, QZ, YQY, and XC). We will download the texts of the potential records to review them for inclusion further. Disagreements will be settled by discussing or consulting a third reviewer (LG and BSD). Figure 1 shows the flow chart of the study selection procedure.

#### 2.3.3 Data extraction and management

Data will be extracted in a pre-designed form of data extraction with Microsoft Excel 2019 (Microsoft, Redmond, WA, www.Microsoft.Com) by teams of 2 reviewers (MXC, SL, QZ, YQY, and XC). Data including their study characteristics (e.g., year of publication, surname of the first author, country where the research was made, reference standards, applied index tests), patient characteristics (sample size, male/female, average age, diagnostic approach used, duration of the interventions) and results (TP, FP, FN, TN) will be collected. Conflicts will be settled by reaching a consensus or consulting a third reviewer (LG and BSD).

### 2.4 Quality evaluation

Applying the standards adapted from the Quality Assessment of Diagnostic Accuracy Studies 2 (QUADAS-2), which is designed to assess the quality of primary diagnostic accuracy studies.^[27]^ The QUADAS-2 tool consists of 4 key domains that discuss patient selection, index test, reference standard, and flow of patients through the study and timing of the index tests and reference standard. The tool is completed in 4 phases: report the review question, develop review specific guidance, review the published flow diagram for the primary study or construct a flow diagram if none is reported, and judge bias and applicability. Each domain is assessed in terms of the risk of bias, and the first 3 domains are also assessed in terms of concerns about applicability. The bias risk for each study will be graded by teams of 2 reviewers (QZ, SL, MXC, YQY, and XC) as low, moderate or high independently. Conflicts will be settled by negotiation. Unified results will be solved by consulting a third reviewer (LG).

### 2.5 Statistical analysis

#### 2.5.1 Meta-analysis

The calculation of pooled sensitivity (SEN), specificity (SPE), negative likelihood ratio, positive likelihood ratio, and diagnostic odds ratio (DOR) will be made by conducting a pairwise meta-analysis with a bivariate mixed-effects regression model in MetaDiSC ver 1.4 (Unit of Clinical Biostatistics Team of the Ramón y Cajal Hospital, Madrid, Spain). Result reports will be within a confidence interval of 95% (95% CI). The heterogeneity among studies will be evaluated by using the inconsistency index (*I*^2^ test; the values of 25%, 50%, and 75% *I*^2^ respectively stood for low, moderate, and high statistical heterogeneity) and the Q value.^[28]^ We will investigate publication bias by using STATA 15.1 (Stata Corporation, College Station, TX) with the program midas.^[29]^ Potential heterogeneity sources will be further researched by the analyses of subgroup and meta-regression. A priori variables that selected as potential sources of heterogeneity will be the study design, reference standard, funding, and study quality.

#### 2.5.2 Quality of evidence

In a conclusive table, the evidence will be graded as “high,” “moderate,” “low,” or “very low” by us with the Grading of Recommendations Assessment, Development and Evaluation (GRADE) approach.^[30]^ The factors that may decrease the quality of the evidence are the study design and risk of bias, inconsistency of the results, indirectness (not generalizable), imprecision (sparse data), and others (e.g., reporting bias). The quality of the evidence for a specific outcome will be reduced by a level on the basis of the performance of the studies against these 5 factors.

## 3 Discussion

For ED patients, the early detection of delirium plays a significant role. A valid screening method will help patients decrease hospitalization time, lower the rate of hospital-acquired complications, and increase life quality after discharge. Existing systematic reviews have confirmed the need for delirium screening in the ED. More and more tools have been developed to facilitate delirium screening for emergency workers, which increase the screening rate for delirium. As the first meta-analysis, the research will assess the accuracy of different screening methods for delirium in ED patients. We will assess the risk of bias of the individual studies, which increased the validity of our conclusions. The results of the final meta-analysis will provide a detailed summary of the evidence of existing screening tools, which will benefit the researchers and policymakers who are interested in delirium screening. However, this study may also have some potential limitations. First, there may be a risk of heterogeneity in the selection of reference standards, examiners and the quality of studies. Second, the reliability of the results largely depends on the comprehensiveness and methodological quality of the main studies included in this review.

## Author contributions

**Data curation:** Qian Zhang, Meixi Chen, Qiuyu Yang, Xiao Cao, Sheng Li.

**Formal analysis:** Meixi Chen, Qian Zhang.

**Methodology:** Qian Zhang, Long Ge, Baoshan Di.

**Project administration:** Qian Zhang, Sheng Li.

**Supervision:** Long Ge, Baoshan Di.

**Validation:** Meixi Chen, Qian Zhang.

**Writing – original draft:** Qian Zhang, Meixi Chen.

**Writing – review & editing:** Qian Zhang, Sheng Li, Long Ge, Baoshan Di.
